# The ratio of shear to elastic modulus of in-plane loaded masonry

**DOI:** 10.1617/s11527-020-01464-1

**Published:** 2020-04-15

**Authors:** Bastian Valentin Wilding, Michele Godio, Katrin Beyer

**Affiliations:** Laboratory of Earthquake Engineering and Structural Dynamics (EESD), School of Architecture, Civil and Environmental Engineering (ENAC), École Polytechnique Fédérale de Lausanne (EPFL), EPFL ENAC IIC EESD, GC B2 495, Station 18, 1015 Lausanne, Switzerland

**Keywords:** Masonry, Shear modulus, Elastic modulus, *G*/*E* ratio, Homogenization

## Abstract

When designing unreinforced masonry buildings, the wall stiffness and, consequently, the masonry elastic and shear modulus *E* and *G* are essential parameters. Current codes provide empirical estimates of the masonry elastic modulus and a ratio between the shear and elastic modulus, *G*/*E*. This ratio, commonly taken as 0.4, is not based on scientific evidence and there appears to be no consensus concerning its value and influencing parameters, meaning that current code standards might not accurately portray the shear deformations of masonry elements. To give the choice of the *G/E* ratio a theoretical foundation, this paper presents closed-form expressions for the *G/E* ratio of running-bond masonry that capture the effects of finite joint thickness, finite wall thickness and orthotropic block properties. Based on the geometry of blocks and joints as well as their elastic parameters, a validation of the developed expression using 3D finite element analyses shows good performance. For modern masonry typologies with hollow clay bricks, a *G*/*E* ratio of 0.20–0.25 is obtained. For historical masonry typologies, such as dry stacked or mortared stone masonry, as well as solid clay brick masonry, ratios between 0.30 and 0.40 are computed.

## Introduction

Unreinforced masonry buildings typically feature a large number of walls and are therefore highly redundant structures. The force distribution in such systems is governed by the in-plane wall stiffness. This parameter is also important in the seismic design of unreinforced masonry buildings, as it strongly influences the dynamic properties of the structure. Overall, the stiffness depends on the wall geometry, the wall static and kinematic boundary conditions, and the elastic properties of the masonry, namely the elastic, or Young’s, modulus *E* and the shear modulus *G*. While it is rather straightforward to determine the wall geometry and the loading conditions, the masonry elastic modulus and, to an even greater extent, the shear modulus, are affected by significant uncertainties. To facilitate engineering work, design codes generally provide estimates for the elastic modulus and recommend the shear modulus be taken as 40% of the elastic modulus, i.e. $G/E = 0.4$ [1–5]. The reported ratio, however, is mainly based on historic practice rather than scientific evidence [5]. In fact, no experimental evidence exists to support this ratio [5], and there is a lack of consensus in the scientific community on its value and its influencing parameters [6, 7]. Authors of in-plane wall tests instead suggest values for *G*/*E* ranging from 0.1 to 0.25 [7, 8].

This paper aims at reducing the uncertainty surrounding the *G*/*E* ratio by providing closed-form expressions of this ratio for in-plane loaded masonry walls with running-bond brick or block work. After comparing the *G*/*E* values recommended in current codes and the literature (Sect. 2) and reviewing existing closed-form expressions from the literature for the elastic properties of masonry (Sect. 3), we conclude that the expression for the *G*/*E* ratio should consider the following points: (1) The deformation of the blocks along with the finite and varying thicknesses of the head and bed joints generally found in mortared brick and stone masonry walls, which are not able to be precisely described by commonly employed ‘interface-block’ models for masonry; (2) The influence of the out-of-plane deformation on the elastic properties of the in-plane loaded masonry wall, or ‘3D effect’, which is due to the finite thickness of the wall and which 2D plane stress conditions often assumed in the literature do not captured correctly; and (3) The orthotropic properties of modern vertically-perforated bricks, which are shown to affect the *G*/*E* ratio and existing models do not consider.

Based on these observations, closed-form expressions for *E*, *G* and the *G*/*E* ratio have been developed. To consider the effect of the finite wall thickness and orthotropic blocks on the masonry elastic properties, a homogenization technique previously employed for periodic running-bond masonry walls under plane stress conditions and with deformable blocks and joints of finite thickness [9] was adopted and extended (Sect. 4). The use of orthotropic blocks is a novelty in the application of homogenization to masonry that allows for a more accurate analysis of modern hollow brick and block masonry. The obtained expressions were validated with 3D finite element (FEM) simulations and compared to other existing formulations by conducting a parametric study on the influence of the geometric and elastic properties of blocks and joints on the *G*/*E* ratio (Sect. 5). Additionally, the influence of orthotropic blocks and of unfilled head joints was studied (Sect. 6). Equations linking the block orthotropic properties to the block void ratio were derived and fed back into the analytical expression obtained for *G*/*E* to study their influence on masonry properties. The influence of unfilled head joints on the *G*/*E* ratio was investigated by expressing it in terms of the head-to-bed joint elastic modulus ratio. Finally, recommendations for *G*/*E* ratios of commonly used masonry typologies to be used in practical applications were derived from the obtained expressions (Sect. 7).

## Codes and literature

**Fig. 1:**
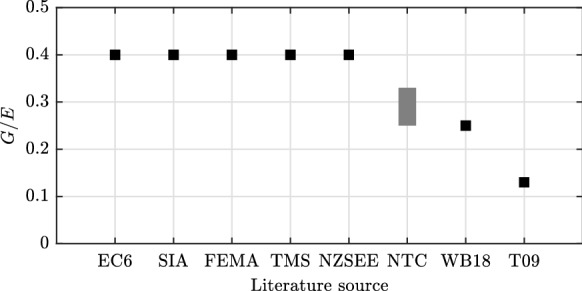
Recommendations for the *G*/*E* ratio according to Part 1 of Eurocode 6 [1], the Swiss standard SIA 266 [4], the pre-standard FEMA 356 [2], the American masonry code TMS 402 [5], the New Zealand guidelines NZSEE [3], the Italian technical standard NTC 08 [11], Wilding and Beyer (WB18) [12], and Tomaševič (T09) [7]

Figure 1 sums up the values for the ratio of shear-to-elastic modulus suggested in codes and in the literature. For modern brick masonry walls, Part 1 of Eurocode 6 [1], the Swiss standard SIA 266 [4], the American masonry code TMS 402 [5], the pre-standard FEMA 356 [2], and the New Zealand seismic guidelines for the assessment of existing buildings NZSEE [3] all propose a *G*/*E* ratio of 0.4. The commentary [10] to the Italian technical standard NTC 08 [11] does not provide explicit values for *G*/*E*, but gives ranges of values for *E* and *G* depending on the masonry typology. Dividing the minimum and maximum values per typology leads to *G*/*E* ratios between 0.29 and 0.33 for dressed regular stone masonry, a ratio of 0.33 for solid brick masonry with lime mortar, of 0.25 for solid brick masonry with cement mortar, and a ratio of 0.30 for vertically-perforated bricks, both with filled and unfilled head joints.

In the literature, the mentioned ratio of 0.4 was already put into question by e.g. Mojsilovic [6] and Tomaševič [7]. Mojsilovic [6] highlighted the importance of taking into account the orthotropic elastic properties of masonry in the evaluation of the *G*/*E* ratio and proposed a simplified formula to be used in the practice. Tomaševič [7] suggested a simple approach for estimating the *G*/*E* ratio, which is outlined in the following. If the masonry elastic modulus *E* is determined from masonry compression tests, the shear modulus *G* can be retrieved from shear-compression tests by using the Timoshenko beam theory with the previously determined elastic modulus and the initial in-plane wall stiffness as inputs. Following this approach for five tests and using an effective wall stiffness, i.e. the secant wall stiffness passing by the point corresponding to the first onset of masonry cracking, a *G*/*E* ratio of 0.1 was proposed [7]. Wilding and Beyer [12] applied this approach to six walls tested by Petry and Beyer [13], using the wall stiffness between 5% and 15% the wall peak force, which is more representative of the wall initial stiffness, and obtained for five walls values around 0.25 and for one wall a value of 0.6. By applying a factor of 0.75 between the effective and the initial wall stiffness for modern hollow clay brick masonry [12], the *G*/*E* ratio proposed by Tomaševič [7] would correspond to 0.13 for the initial masonry properties. Petry and Beyer [8] used a *G*/*E* ratio of 0.25 in the validation of their beam element model.

## Existing closed-form expressions for masonry elastic properties

The number of works to be found in the literature on obtaining the equivalent in-plane elastic properties of masonry by means of homogenization, or averaging, methods is significant, e.g. [14–25]. Many of them, however, model the masonry as an assembly of discrete blocks and interfaces. Only very few works report closed-form expressions for *E* and *G* considering the blocks as deformable and the mortar joints of finite thickness, see Taliercio [9] and the references contained therein. Among these few works, those of Pande et al. [26], Cecchi and Sab [27, 28], and Taliercio [9] are selected for comparison with the expressions derived in this paper (Sect. 5).

The work by Pande et al. [26] is selected since it provides a simple two-step averaging procedure for deriving the properties of masonry in closed-form both under 2D plane stress and 3D stress conditions. The masonry wall was approximated as a superposition of layers stacked in the two in-plane wall directions. In the first step, average properties of the layer representing the blocks and the layer representing the mortar head joints were derived. In the second step, a system containing alternating horizontal layers of the equivalent material derived from the first step of the procedure and bed joint mortar was solved in order to obtain the masonry elastic properties. The expressions obtained through this procedure were shown to depend on the order of averaging the stacked layers [29]. In this paper, only the original order of the steps is considered. Its performance in predicting the masonry properties under plane stress conditions has already been investigated [9].

Cecchi and Sab [27] derived closed-form expressions of the masonry elastic properties by means of an asymptotic homogenization technique. In their work, the homogenization problem was expressed and solved as a function of three parameters: a scale parameter expressing the ratio between the bed joint thickness and the block height, a parameter herein called ‘contrast’, representing the ratio between the elastic moduli of mortar and blocks, and a further parameter relative to the aspect ratio of the blocks. Both plane strain and plane stress states were considered for the wall and analytical closed-form expressions for *E* and *G* were provided based on the assumption that the mortar joints are infinitely thin compared to the block height. Cecchi and Sab [28], extended the above technique to capture the effect of the finite thickness of the wall on the masonry elastic properties by considering blocks in plane stress state and attributing a plane strain condition to the mortar, see also Cecchi et al. [30]. The expressions derived in this paper rely on the same hypothesis with regard to the stress-deformation state of block and mortar (Sect. 4) and, for this reason, the model by Cecchi and Sab [27, 28] is considered for comparison.

Taliercio [9] derived closed-form expressions for the masonry elastic properties of walls by using an approach derived from Aboudi’s method of cells [31] in conjunction with the homogenization theory. The approach consists in subdividing the representative volume element (RVE) of the masonry wall into several sub-domains, or cells, and assuming polynomial functions describing the displacement field inside each cell. The homogenization problem is formulated by applying to the RVE a compression and shear deformation and computing the resulting stresses. From these, analytical expressions for *E* and *G* are retrieved. Taliercio used two approaches to solve the homogenization problem: the first, derived from the original method of cells [31], consisted in solving the problem by enforcing continuity of the stresses between the cells; the second, in minimizing the potential energy of the representative volume. Only the second approach will be used for comparison, as it was found to give more accurate results [9].

With respect to the above mentioned works [26, 28], in [9] masonry was modeled under a 2D plane stress condition. As shown in Sect. 4, the formulations developed in this paper are built on Taliercio’s approach [9]. For this reason, its expressions are evaluated in the following for comparison. Taliercio’s expressions are extended to capture the effect of the finite wall thickness on the masonry elastic properties. This is done by replacing the plane stress state [9], and the generalized plane strain state of the original method of the cells [31], by different assumptions with regard to the stress-deformation conditions for the in-plane loaded masonry. In addition, new formulations are introduced in order to capture the influence of orthotropic blocks on the masonry elastic properties.

## Formulation of the *G*/*E* ratio

Expressions for the elastic properties *E* and *G* along with the *G*/*E* ratio of masonry are developed by taking into account the effect (1) of the deformation of the blocks and the finite thickness of the mortar joints, which can be different between the head and the bed joints, (2) of the finite wall thickness, or ‘3D effect’, and (3) of the orthotropic properties of the blocks on the elastic properties of the in-plane loaded masonry. The formulation builds on the homogenization technique proposed by Taliercio [9], but differs from this latter with regard to the stress-deformation state assumed inside the in-plane loaded wall (Sect. 4.1) and the consideration of orthotropic block properties in the model (Sects. 4.2, 4.3).

### Assumption on the stress-deformation state

When subjected to in-plane loading, masonry walls undergo both in-plane and out-of-plane deformations [29]. In masonry buildings the thickness of the walls is far from being small (or large) compared to the size of the blocks [32] and therefore none of the hypotheses of plane stress (or plane strain) state applies a priori to the wall middle plane [32–35]. In other words, the stress-deformation state in masonry can neither be accurately expressed using solely a plane stress nor only a plane strain hypothesis for wall thicknesses used in the practice. In order to seize the effect of the finite thickness of the wall on its in-plane behaviour, several research groups developed approaches taking into account the actual 3D stress-deformation state of wall [17, 19, 25, 35], or put forward descriptions based on a generalized plane strain state on the wall middle plane [32–34, 36–38].

**Fig. 2:**
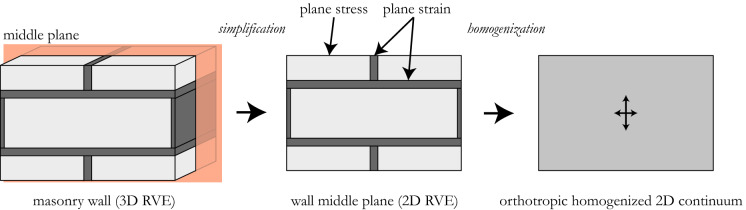
Procedure applied for deriving the equivalent elastic properties of an in-plane loaded masonry wall

The formulation proposed here builds on the hypothesis of a plane stress state inside the blocks and a plane strain state inside the joints. This assumption allows mimicking the stress-deformation state that is found in the wall middle plane (Fig. 2), without the need of introducing the above mentioned 3D or generalized plane descriptions, which all add new variables to the homogenization problem to be solved. In [28, 30], this assumption was already used while considering the joints as interfaces. In the present work, the applicability of this approach is extended to joints of finite thickness.

Observations based on finite element simulations [29, 32, 33, 35, 39] and experimental results on masonry under compression [40, 41] further justify this assumption. Under in-plane vertical loading, the mortar tends to expand more in the out-of-plane direction than the block, the Poisson’s ratio of the former being larger than the one of the latter. The bond between mortar and the block, however, prevents the mortar expansion, which results in a tri-axial compression state inside the mortar and a bi-lateral tension along with a vertical compression inside the block. This state of lateral confinement in the joints may be modelled by means of a plane strain condition, while a plane stress condition applies for the blocks [28, 30].

As illustrated in Fig. 2, solving the homogenization problem not for the 3D representative volume element (RVE) of the wall, but for the 2D RVE of the wall middle plane, represents a simplification in view of the homogenization process, which, strictly speaking, should start from a 3D RVE [27, 28, 35]. Considering only the middle plane of the wall (Fig. 2), equilibrium and compatibility conditions hold true even if different stress-deformation states are assumed in blocks and joints. In fact, for reasons of symmetry, under in-plane loading conditions the wall does not develop out-of-plane displacements in its middle plane, neither in plane strain nor the plane stress conditions. In the following, it will be shown, by benchmarking the results against 3D finite element simulations, that the assumption made here with regard to the stress-deformation state of the wall middle plane leads to a very good estimation of the masonry elastic properties.

### Derivation of the masonry elastic modulus *E*

**Fig. 3:**
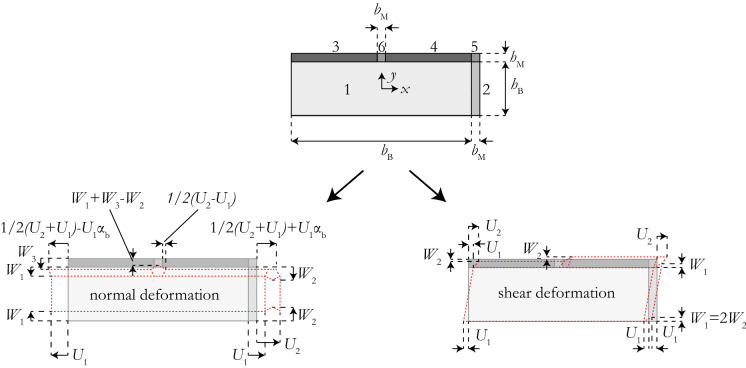
Division of the 2D RVE into cells after Taliercio [9] including corresponding normal and shear deformations

Following Taliercio [9], the RVE is divided into six cells representing the block along with the head and bed joints, as shown in Fig. 3. The displacement fields $u_{ix}$ and $u_{iy}$ in the *x*- and *y*-directions inside each cell *i* of the RVE, here denoted as microscopic, are provided by means of polynomial functions describing the deformed shape of the RVE under normal deformation (Fig. 3):

1 $$\begin{aligned} \begin{aligned}&u_{1x} = 2U_1\frac{x}{b_{\rm B}} \\&u_{1y} = -2W_1\frac{y}{h_{\rm B}} \\&u_{2x} = U_1+\frac{\left( U_2-U_1\right) \left( x-\frac{b_{\rm B}}{2}\right) }{b_{\rm M}}\\&u_{2y} = -2\frac{y}{h_{\rm B}}\left( \frac{2\left( W_1-W_2\right) \left| \frac{b_{\rm M}+b_{\rm B}}{2}-x \right| }{b_{\rm M}}+W_2\right) \\&u_{3x} = u_{1x}-\frac{\left( U_1\left( 1+2\frac{b_{\rm M}}{b_{\rm B}}\right) -U_2\right) \left( \frac{h_{\rm B}}{2}-y\right) }{2h_{\rm M}}\\&u_{3y} = -W_1+\frac{\left( W_1-W_3\right) \left( y-\frac{h_{\rm B}}{2}\right) }{h_{\rm M}} \\&u_{4x} = u_{1x}+\frac{\left( U_1\left( 1+2\frac{b_{\rm M}}{b_{\rm B}}\right) -U_2\right) \left( \frac{h_{\rm B}}{2}-y\right) }{2h_{\rm M}} \\&u_{4y} = u_{3y} \\&u_{5x} = -\frac{\left( {y}-\frac{ {h_{\rm B}}}{2}\right) \left( \frac{ {b_{\rm B}}+ {b_{\rm M}}}{2}- {x}\right) \left( {U_1} \left( \frac{2 {b_{\rm M}}}{ {b_{\rm B}}}+1\right) - {U_2}\right) }{ {b_{\rm M}} {h_{\rm M}}} \\& \quad \qquad -\,\frac{\left( {x}-\frac{ {b_{\rm B}}}{2}\right) ( {U_1}- {U_2})}{ {b_{\rm M}}}+ {U_1} \\&u_{5y} = -W_3\frac{y-\frac{h_{\rm B}}{2}}{h_{\rm M}} \\& \quad \qquad -2\frac{\left( W_2\frac{b_{\rm M}}{2}-\left( W_2-W_1\right) \left| \frac{b_{\rm B}+b_{\rm M}}{2}-x\right| \right) \left( \frac{h_{\rm B}}{2}+h_{\rm M}-y\right) }{b_{\rm M} h_{\rm M}} \\&u_{6x} = \frac{2 {x}}{ {b_{\rm B}}} \left( {U_1}-\frac{\left( {y}-\frac{ {h_{\rm B}}}{2}\right) \left( \frac{ {b_{\rm B}} ( {U_1}- {U_2})}{2 {b_{\rm M}}}+ {U_1}\right) }{ {h_{\rm M}}}\right) \\&u_{6y} = -W_1+\frac{\left( W_2-W_3+2\left( W_1-W_2\right) \frac{|x|}{b_{\rm M}}\right) \left( y-\frac{h_{\rm B}}{2}\right) }{h_{\rm M}}. \end{aligned} \end{aligned}$$

Taliercio [9] derived these functions, featuring the polynomial coefficients $U_1$, $U_2$, $W_1$, $W_2$, $W_3$, and the quantities $b_{\rm B}$, $b_{\rm M}$, $h_{\rm B}$, $h_{\rm M}$ capturing the block (B) and mortar joint (M) geometry (Fig. 3).

The microscopic strains $\varepsilon _{ixx}$, $\varepsilon _{iyy}$, $\varepsilon _{ixy}$ inside each cell can be obtained by differentiating the corresponding microscopic displacement field. The resulting microscopic stresses inside the cells of the RVE corresponding to mortar ($i=2,\ldots ,6$) can be derived by assigning an isotropic linear elastic constitutive law to the the mortar:

2 $$\begin{aligned} \begin{aligned}&\sigma _{ixx} = \left( \lambda _{\rm M}+2\mu _{\rm M}\right) \varepsilon _{ixx}+\lambda _{\rm M}\varepsilon _{iyy} \\&\sigma _{iyy} = \lambda _{\rm M}\varepsilon _{ixx} + \left( \lambda _{\rm M}+2\mu _{\rm M}\right) \varepsilon _{iyy} \\&\sigma _{ixy} = 2\mu _{\rm M}\varepsilon _{ixy}, \end{aligned} \end{aligned}$$

and by assuming the Lamé constants for a plane strain state:

3 $$\begin{aligned} \begin{aligned}&\lambda _{\rm M} = \frac{\nu _{\rm M}{E_{\rm M}}}{(1+\nu _{\rm M})(1-2\nu _{\rm M})} \\&\mu _{\rm M} = \frac{E_{\rm M}}{{2\left( {1 + \nu _{\rm M} } \right) }}, \end{aligned} \end{aligned}$$

where $E_{\rm M}$ is the mortar elastic modulus and $\nu _{\rm M}$ the mortar Poisson’s ratio. The block is modelled as orthotropic and its elastic properties are thus defined by the elastic moduli in the horizontal and vertical directions, $E_{\rm B,1}$ and $E_{\rm B,2}$, the Poisson’s ratios $\nu _{\rm B,12}$, $\nu _{\rm B,21}$, and the shear modulus, $G_{\rm B}$. The shear modulus cannot be directly related to the elastic moduli, as in the isotropic case, and only the following relationship holds: $E_{\rm B,1}/\nu _{\rm B,12}=E_{\rm B,2}/\nu _{\rm B,21}$. Assigning a plane stress state inside the block leads to the following stresses in cell 1 of the RVE:

4 $$\begin{aligned} \begin{aligned}&\sigma _{1xx} = \frac{E_{\rm B,1}}{1-\nu _{\rm B,12}\nu _{\rm B,21}} \left( \varepsilon _{1xx} + \nu _{\rm B,21}\varepsilon _{1yy}\right) \\&\sigma _{1yy} = \frac{E_{\rm B,2}}{1-\nu _{\rm B,21}\nu _{\rm B,12}} \left( \nu _{\rm B,12} \varepsilon _{1xx} + \varepsilon _{1yy} \right) \\&\sigma _{1xy} = 2G_{\rm B} \varepsilon _{1xy}. \end{aligned} \end{aligned}$$

The macroscopic strains $E_{xx}$, $E_{yy}$ and $E_{xy}$ computed on the RVE are obtained by integrating the microscopic ones over the RVE volume *V* and result in [9]:

5 $$\begin{aligned} \begin{aligned}&E_{xx} = \frac{1}{V}\int _{V} \varepsilon _{xx} dV = \frac{U_1+U_2}{b_{\rm B}+b_{\rm M}} \\&E_{yy} = \frac{1}{V}\int _{V} \varepsilon _{yy} dV = -\frac{W_1+W_3}{h_{\rm B}+h_{\rm M}} \\&E_{xy} = \frac{1}{V}\int _{V} \varepsilon _{xy} dV = 0. \end{aligned} \end{aligned}$$

Setting $W_2 = W_1$ so that the stresses and strains are uniform over the cells of the RVE except for cells 5 and 6 [9], defining the difference between the potential energy $\pi _m$ stored at the microscopic level and the energy $\pi _M$ stored at the macroscopic level as

6 $$\begin{aligned} \begin{aligned} \varPi&= \frac{1}{2V}\int _{V}^{}\left( \varepsilon _{xx}\sigma _{xx}+2\varepsilon _{xy}\sigma _{xy}+\varepsilon _{yy}\sigma _{yy}\right) \text {d}V \\& \quad \qquad - \left( E_{xx}\varSigma _{xx}+2E_{xy}\varSigma _{xy}+E_{yy}\varSigma _{yy}\right) , \end{aligned} \end{aligned}$$

and differentiating this latter with respect to the remaining coefficients $U_1$, $U_2$, $W_1$, $W_3$ leads to expressions including the unknown macroscopic stresses $\varSigma _{xx}$, $\varSigma _{yy}$, $\varSigma _{xy}$ [9]. Plugging these expressions into Eq. 5 results in relationships between the macroscopic stresses and strains. The equivalent, or homogenized, elastic moduli of masonry *E*, in the direction perpendicular to the bed joints ($y-$direction), and $E_h$, in the direction perpendicular to the head joints ($x-$direction), are retrieved from said equations by imposing a uni-axial stress state on the RVE:

7 $$\begin{aligned} \begin{aligned}&E_{xx} = \frac{1}{E_h} \varSigma _{xx}, \quad \text {for } \varSigma _{yy} = 0 \\&E_{yy} = \frac{1}{E} \varSigma _{yy}, \quad \text {for } \varSigma _{xx} = 0. \end{aligned} \end{aligned}$$

Solving the equations for the elastic moduli *E* and $E_h$, the following closed-form expressions are obtained:

8 $$\begin{aligned} E = \frac{2 {h} ({h_{\rm B}}+{h_{\rm M}})^2}{b \left( a (c+3 d) - (e-f)^2\right) -g^2 (c+3 d)} \end{aligned}$$

and

9 $$\begin{aligned} E_h = \frac{2 {h} ({b_{\rm B}}+{b_{\rm M}})^2}{d \left( (c-d) (a+b-2 g) - (e-f)^2\right) }, \end{aligned}$$

where:

10 $$\begin{aligned} \begin{aligned} h &= a \left( b d (c-d)-e^2 (c+3 d)\right) \\& \quad - b \left( c e^2+d f (2 e+f)\right) +2 e g (e (c+d)+2 d f) \\& \quad - d g^2 (c-d)+e^2 (e-f)^2 \end{aligned} \end{aligned}$$

and

11 $$\begin{aligned}&a = -\frac{96 \alpha _{\rm b} \alpha _{\rm B}^{2} \alpha _{\rm EB} \alpha _{\rm h} E_{{\rm{B}},2} \left( 2 \nu _{\rm M}^{2}+ \nu _{\rm M}-1\right) + \alpha _{\rm E} E_{{\rm{B}},2} \left( \alpha _{\rm EB} \nu _{{\rm{B}},21}^{2}-1\right) \left( \alpha _{\rm b}^{4} (8 \nu _{\rm M}-4)+ \alpha _{\rm b}^{3} (8-16 \nu _{\rm M})+ \alpha _{\rm b}^{2} \left( 32 \alpha _{\rm B}^{2} \alpha _{\rm h}^{2} ( \nu _{\rm M}-1)-22 \nu _{\rm M}+11\right) + \alpha _{\rm b} \left( -64 \alpha _{\rm B}^{2} \alpha _{\rm h}^{2} ( \nu _{\rm M}-1)-6 \nu _{\rm M}+3\right) -8 \alpha _{\rm B}^{2} \alpha _{\rm h} (2 \alpha _{\rm h}+3) ( \nu _{\rm M}-1)\right) }{48 \alpha _{\rm b} ( \alpha _{\rm b}+1) \alpha _{\rm h} ( \alpha _{\rm h}+1) h_{\rm B}^{2} ( \nu _{\rm M}+1) (2 \nu _{\rm M}-1) \left( \alpha _{\rm EB} \nu _{{\rm{B}}21}^{2}-1\right) } \nonumber \\&b = -\frac{ \alpha _{\rm E} E_{{\rm{B}},2} \left( \alpha _{\rm b}^{2} (2 \nu _{\rm M}-1)+ \alpha _{\rm b} (3-6 \nu _{\rm M})-8 \alpha _{\rm B}^{2} \alpha _{\rm h} (2 
\alpha _{\rm h}+3) ( \nu _{\rm M}-1)\right) }{48 \alpha _{\rm b} ( \alpha _{\rm b}+1) \alpha _{\rm h} ( \alpha _{\rm h}+1) h_{\rm B}^{2} ( \nu _{\rm M}+1) (2 \nu _{\rm M}-1)} \nonumber \\&c = \frac{ E_{{\rm{B}},2} \left( \alpha _{\rm E} (\nu _{\rm M}-1) (4 \alpha _{\rm b} \alpha _{\rm h}+\alpha _{\rm b}+1) \left( \alpha _{\rm EB} \nu _{{\rm{B}},21}^{2}-1\right) -4 \alpha _{\rm H} \left( 2 \nu _{\rm M}^{2}+\nu _{\rm M}-1\right) \right) }{2 ( \alpha _{\rm b}+1) \alpha _{\rm h} ( \alpha _{\rm h}+1) h_{\rm B}^{2} ( \nu _{\rm M}+1) (2 \nu _{\rm M}-1) \left( \alpha _{\rm EB} \nu _{{\rm{B}},21}^{2}-1 \right) } \nonumber \\&d = \frac{ \alpha _{\rm E} E_{{\rm{B}},2} ( {\nu _{\rm M}}-1)}{2 {\alpha _{\rm h}} ( {\alpha _{\rm h}}+1) h_{\rm B}^{2} ( {\nu _{\rm M}}+1) (2 {\nu _{\rm M}}-1)} \nonumber \\&e = \frac{ {\alpha _{\rm B}} \alpha _{\rm E} E_{{\rm{B}},2} {\nu _{\rm M}}}{2 ( {\alpha _{\rm b}}+1) ( {\alpha _{\rm h}}+1) h_{\rm B}^{2} ( {\nu _{\rm M}}+1) (2 {\nu _{\rm M}}-1)} \nonumber \\&f = \frac{ - \alpha _{\rm B} E_{{\rm{B}},2} \left( \alpha _{\rm EB} \nu _{{\rm{B}},21} \left( \nu _{\rm M} (3 \alpha _{\rm E} \nu _{{\rm{B}},21}-4)-8 \nu _{\rm M}^{2}+4\right) -3 \alpha _{\rm E} \nu _{\rm M}\right) }{2 ( {\alpha _{\rm b}}+1) ( {\alpha _{\rm h}}+1) h_{\rm B}^{2} ( {\nu _{\rm M}}+1) (2 {\nu _{\rm M}}-1) \left( {\alpha _{\rm EB}} \nu _{{\rm{B}},21}^{2}-1\right) } \nonumber \\&g = \frac{ \alpha _{\rm E} E_{{\rm{B}},2} \left( \alpha _{\rm b}^{3} (4 {\nu _{\rm M}}-2)+ \alpha _{\rm b}^{2} (5-10 {\nu _{\rm M}})+ {\alpha _{\rm b}} \left( 16 \alpha _{\rm B}^{2} \alpha _{\rm h}^{2} ( {\nu _{\rm M}}-1)-6 {\nu _{\rm M}}+3\right) -8 \alpha _{\rm B}^{2} {\alpha _{\rm h}} (2 {\alpha _{\rm h}}+3) ( {\nu _{\rm M}}-1)\right) }{48 {\alpha _{\rm b}} ( {\alpha _{\rm b}}+1) {\alpha _{\rm h}} ( {\alpha _{\rm h}}+1) h_{\rm B}^{2} ( {\nu _{\rm M}}+1) (2 {\nu _{\rm M}}-1)}. \end{aligned}$$

The parameters denoted with $\alpha$ entering the above expressions are scale parameters [27] governing the homogenized masonry properties:

12 $$\begin{aligned} &\alpha _{\rm B} = \frac{ h_{\rm B}}{ b_{\rm B}}\quad \alpha _{\rm M} = \frac{ h_{\rm M}}{ b_{\rm M}} \\&\alpha _{\rm b} = \frac{ b_{\rm M}}{ b_{\rm B}}\quad \alpha _{\rm h} = \frac{ h_{\rm M}}{ h_{\rm B}} \\&\alpha _{\rm G} = \frac{ G_{\rm M}}{ G_{\rm B}}\quad \alpha _{\rm E} = \frac{ E_{\rm M}}{ E_{\rm B,2}} \\&\alpha _{\rm EB} = \frac{ E_{\rm B,1}}{ E_{\rm B,2}}. \end{aligned}$$

The first four parameters, $\alpha _{\rm B},\alpha _{\rm M},\alpha _{\rm b},\alpha _{\rm h}$, are related to the geometry of the RVE, whereas the last three, $\alpha _{\rm G},\alpha _{\rm E},\alpha _{\rm EB}$, are related to the material properties of the block and the mortar. The first six parameters are defined as in [9]; $\alpha _{\rm EB}$ is an additional parameter herein introduced to capture the orthotropy of the blocks.

### Derivation of the masonry shear modulus *G*

The procedure for deriving the shear modulus of masonry *G* is the same as for the derivation of the elastic moduli *E* and $E_h$. A macroscopic shear deformation is reproduced by the microscopic displacement field inside the RVE shown in Fig. 3, and its form is given by [9]:

13 $$\begin{aligned} \begin{aligned}&u_{1x} = \frac{2 {U_1} {y}}{ {h_{\rm B}}} \\&u_{1y} = 0 \\&u_{2x} = u_{1x} \\&u_{2y} = \frac{ {W_1} \left( { x}-\frac{ {b_{\rm B}}}{2}\right) }{ {b_{\rm M}}} \\&u_{3x} = \frac{\left( {y}-\frac{ {h_{\rm B}}}{2}\right) ( {U_2}- {U_1})}{ {h_{\rm M}}}+ {U_1} \\&u_{3y} = -\frac{ {W_2} \left( {y}-\frac{ {h_{\rm B}}}{2}\right) }{ {h_{\rm M}}} \\&u_{4x} = u_{3x} \\&u_{4y} = -u_{3y}\\&u_{5x} = u_{3x} \\&u_{5y} = -\frac{ {W_1} \left( \left( {y}-\frac{ {h_{\rm B}}}{2}\right) \left( x-\frac{b_{\rm B} + b_{\rm M}}{2} \right) - {h_{\rm M}} \left( { x}-\frac{ {b_{\rm B}}}{2}\right) \right) }{ {b_{\rm M}} {h_{\rm M}}} \\&u_{6x} = u_{3x} \\&u_{6y} = \frac{ {W_1} { x} \left( {y}-\frac{ {h_{\rm B}}}{2}\right) }{ {b_{\rm M}} {h_{\rm M}}}. \end{aligned} \end{aligned}$$

The microscopic strains $\varepsilon _{ixy}$, microscopic stresses $\sigma _{ixy}$, macroscopic strains $E_{xy}$ and the potential energies $\pi _m$ and $\pi _M$ are obtained in the same manner as when computing the elastic moduli (see Eqs. 2–6). For the macroscopic shear deformation, the macroscopic strains are:

14 $$\begin{aligned} \begin{aligned} E_{xx} =&\frac{1}{V}\int _{V}^{} \varepsilon _{xx} dV = 0 \\ E_{yy} =&\frac{1}{V}\int _{V}^{} \varepsilon _{yy} dV = 0 \\ E_{xy} =&\frac{1}{V}\int _{V}^{} \varepsilon _{xy} dV = \frac{U_1+U_2}{2(h_{\rm B}+h_{\rm M})}+\frac{W_2}{b_{\rm B}+b_{\rm M}}. \end{aligned} \end{aligned}$$

Differentiating the difference in potential energy $\varPi$ with respect to the newly defined polynomial coefficients $U_1$, $U_2$, $W_1$ and $W_2$, setting $W_1=2W_2$, inserting the result into Eq. 14 and collecting all the terms around $\varSigma _{xy}$ leads to a closed-form expression for the shear modulus *G*, which reads:

15 $$\begin{aligned} G = \alpha _{\rm G} {G_{\rm B}} \left( 1+ \frac{1-\alpha _{\rm G}}{\alpha _{\rm G}+\alpha _{\rm h} + \alpha _{\rm b} (1 + \alpha _{\rm h}) \left( 1-\frac{(1-{\alpha _{\rm G}}) {p}}{{p}+{q}}\right) } \right) . \end{aligned}$$

The parameters $\alpha$ are defined by Eq. 12 while *p* and *q* are additional parameters defined as

16 $$\begin{aligned} \begin{aligned}&p = \alpha _{\rm h} \left( 3+\alpha _{\rm b} \left( 2-\alpha _{\rm b} \right) \left( 1 + 2 \alpha _{\rm M}^2 \left( 1-\nu _{\rm M}'\right) \right) \right) \\&q = 6 \alpha _{\rm b} \alpha _{\rm M}^2 \left( 1-\nu _{\rm M}'\right) , \end{aligned} \end{aligned}$$

with

$$\begin{aligned} \nu _{\rm M}'= \frac{\nu _{\rm M}}{1-\nu _{\rm M}}. \end{aligned}$$

The derivation shows that the orthotropy of the blocks does not influence the form of the equations but enters only through the shear modulus of the block $G_{\rm B}$ (Sect. 6). Moreover, replacing the term $\nu _{\rm M}'$ in Eq. 16 with $\nu _{\rm M}$, Taliercio’s expression for *G* under plane stress condition is obtained [9].

## Comparison with existing expressions and parametric study on the *G*/*E* ratio

**Fig. 4:**
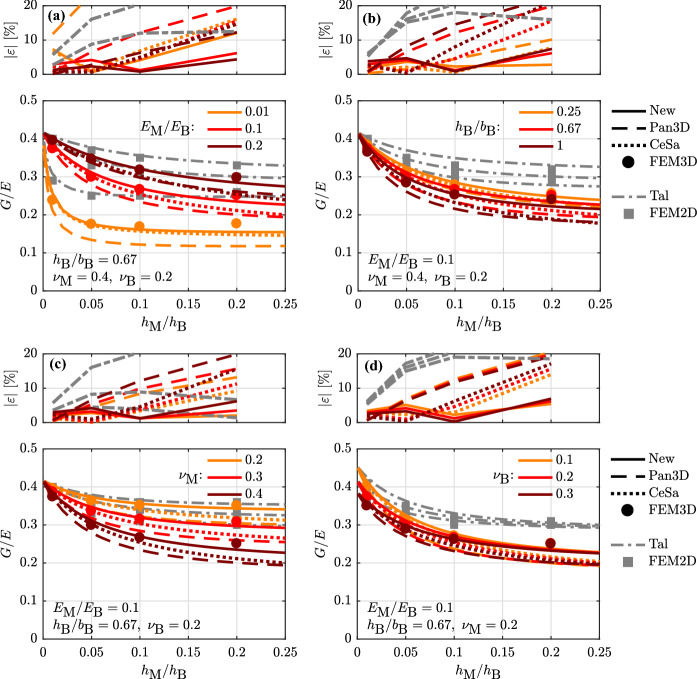
Validation and parametric study on the shear-to-elastic modulus ratio *G*/*E* of masonry for isotropic blocks and head and bed joints of equal thickness. Compared are: 2D and 3D FEM analyses, ‘FEM2D’ and ‘FEM3D’; the expressions by Pande et al. [26], ‘Pan3D’; by Cecchi and Sab [27, 28], ‘CeSa’; by Taliercio [9], ‘Tal’; and the proposed expression, ‘New’

The formulation for the shear-to-elastic modulus ratio *G*/*E* can be obtained by dividing the derived expressions for *E* and *G* (Eqs. 8, 15). In Fig. 4, the performance of the so-obtained formulation for the *G*/*E* ratio of masonry is investigated by comparing it to other expressions from the literature along with the results from 2D and 3D FEM analyses. The expressions considered for comparison are those introduced in Sect. 3: ‘Pan3D’ by Pande et al. [26], ‘CeSa’ by Cecchi and Sab [27, 28], and ‘Tal’ by Taliercio [9]. The blocks are modelled as isotropic to meet the assumption underlying these models. In the newly derived expressions, ‘New’, this is obtained by setting $E_{\rm B,1}=E_{\rm B,2}=E_{\rm B}$ and $\nu _{\rm B,12}=\nu _{\rm B,21}=\nu _{\rm B}$. Moreover, the head and the bed joints are modelled with the same thickness and properties.

The FEM analyses are performed with the software Abaqus using the plug-in for numerical homogenization developed by Omairey et al. [42], suitably modified to apply periodic boundary conditions in the 2 in-plane directions only [29, 35]. Said version of the plug-in has been validated by reproducing the results of the numerical homogenization of a masonry wall presented in [29]. For brevity, the validation is not reported here.

The 2D simulations, ‘FEM2D’, are conducted on a 2D RVE using plane stress conditions. The 3D simulations, ‘FEM3D’, are conducted on a 3D RVE with varying thickness of 100, 200 and 400 mm. In the following, only the results for a wall thickness of 200 mm are shown in the comparison, while supplemental material to the paper reports the results of the analyses for wall thicknesses of 100 mm and 400 mm (see section ‘Reproducibility of the article content’). These analyses show that in the case of relatively thin mortar joints, the performance of the model remains the same, no matter the wall thickness; for relatively thick joints, the model performance increases with increasing wall thickness. Only thicknesses of 200 mm and 400 mm are considered of interest for the practice as 100 mm is below the minimum wall thickness allowed by currently used standards (see e.g. NTC [11]).

In the parametric study, the joint-to-block height ratio $h_{\rm M}/h_{\rm B}$ is varied along with the contrast $E_{\rm M}/E_{\rm B}$ (Fig. 4a), the block aspect ratio $h_{\rm B}/b_{\rm B}$ (Fig. 4b), the joint Poisson’s ratio $\nu _{\rm M}$ (Fig. 4c) and the block Poisson’s ratio $\nu _{\rm B}$ (Fig. 4d), to study their influence on the *G*/*E* ratio. The main influencing parameters appear to be the contrast (a), the joint Poisson’s ratio (c) and, generally, the joint-to-block height ratio $h_{\rm M}/h_{\rm B}$. The *G*/*E* ratio shows a positive tendency with regard to the first parameter, while it is the opposite case for the last two. The block aspect ratio (b) and the block Poisson’s ratio (d) appear to have a significantly smaller effect.

For all studied configurations, there appears to be a significant difference between the *G*/*E* ratio predicted by the 2D and 3D models along with the corresponding FEM analyses. As highlighted in the literature, 2D FEM analyses based on the plane stress assumption give lower values of *E* with respect to the 3D case since they neglect the effect of the wall thickness [29]. On the contrary, 2D and 3D analyses give a shear modulus that is basically the same [29]. This results in higher *G*/*E* ratios for the 2D compared to the 3D analyses. The difference obtained between 2D and 3D analyses was previously observed to be as high as 4% in [29]; a higher discrepancy was shown in [30]. Depending on the masonry configuration (Fig. 4), this difference reaches here 35%, showing that a 2D plane stress condition might not be accurate in describing the stress-deformation state of masonry - even in the linear range (c.f. [29]). As the difference between 2D and 3D analyses is significant for nearly all the investigated parameters, the 2D results are plotted in Fig. 4 continuously in grey, to highlight their secondary nature concerning the parametric study.

The ‘Tal’ model corresponds very well to the results of the 2D FEM analyses, while the ‘New’ model along with the other models, ‘Pan3D’ and ‘CeSa’, are close to the results of the 3D FEM analyses. In order to understand which of the ‘Pan3D’, ‘CeSa’ and ‘New’ expressions performs best in predicting the 3D FEM analyses, the relative error $|\varepsilon |$ is plotted in Fig. 4. This error is defined as the difference between model prediction and ‘FEM3D’ result divided by the ‘FEM3D’ result. The comparison between ‘Tal’, which is formulated for a 2D RVE, and the 3D FEM analyses is included only for sake of the reference. In general, the derived expression ‘New’ shows the best prediction of the *G*/*E* ratio, especially for configurations where the other models show large relative errors, i.e. with increasing joint-to-block height ratio. The relative errors of the other formulations reach, in fact, more than 15% for large $h_{\rm M}/h_{\rm B}$ ratios, while the error of the new expression stays below 7% for all the studied configurations, except for one in which the contrast is very low (Fig. 4a). The agreement of ‘CeSa’ with the 3D FEM analyses improves with decreasing joint-to-block height ratio but reduces with increasing contrast. The different performance of ‘CeSa’ and ‘New’ finds its justification in the modelling of the joints. Although a plane strain condition is used in both models, ‘CeSa’ models the joints as zero-thickness interfaces. ‘Pan3D’ constantly underestimates the *G*/*E* ratio, to a greater extent with increasing $h_{\rm M}/h_{\rm B}$ ratio.

In conclusion, the derived expressions for *E* and *G* (‘New’) capture the effect of the finite joint thickness and successfully extend the work of Taliercio [9] to model the influence of the 3D effect, i.e. the effect of the limited wall thickness on the masonry elastic properties and therefore also on its *G*/*E* ratio.

## Influence of vertically-perforated blocks and unfilled head joints

Modern masonry construction often makes use of vertically-perforated blocks [42–45]. The perforation leads to orthotropic block properties. The influence of the perforation on the block properties is studied in two steps: first, a numerical study varying the perforation pattern and the block void ratio is conducted and empirical equations relating the resulting orthotropic block properties to the block void ratio derived (Sect. 6.1); second, said equations are plugged into the closed-form expressions for *E* and *G* derived in Sect. 4 and the influence of the block void ratio on the masonry elastic properties is studied (Sect. 6.2). As a further point (Sect. 6.3), the influence of unfilled head joints, another practice adopted in modern masonry construction [44], is studied. This is done by gradually changing the ratio of head joint to bed joint elastic modulus ratio.

### Elastic properties of vertically-perforated blocks

**Fig. 5:**
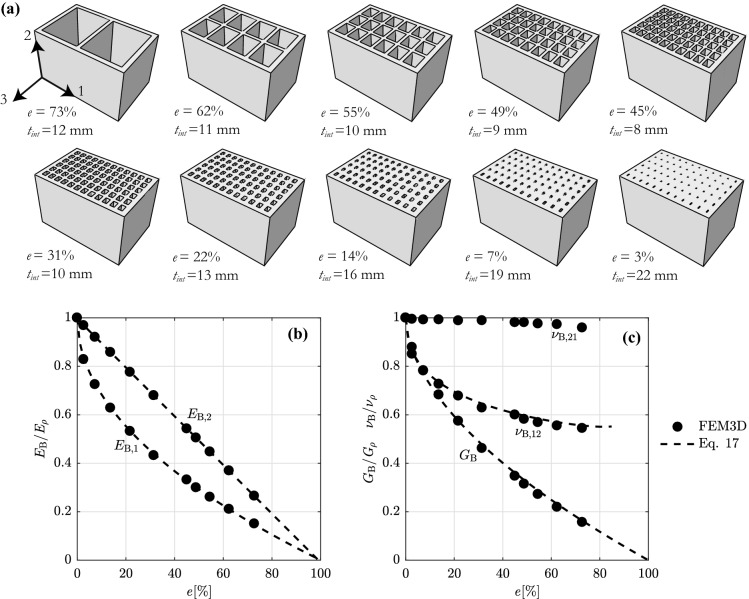
**a** FEM models of vertically-perforated blocks used to study the influence of the block void ratio on the block elastic properties. **b** and **c** Influence of the block void ratio *e* on the block elastic properties. Comparison between 3D FEM analyses and the proposed Eq. 17

The development of the orthotropic elastic block properties $E_{\rm B,1}$, $E_{\rm B,2}$, $G_{\rm B}$, $\nu _{\rm B,12}$ and $\nu _{\rm B,21}$, introduced in Eq. 4, with varying block void ratio is derived by means of FEM analyses conducted in Abaqus on a block model of 190 mm height, 300 mm length and a width of 195 mm (Fig. 5a). The void ratio *e* is defined as the ratio of the volume of voids to the total block volume. The homogenization plug-in by Omairey et al. [42], modified to apply period boundary conditions in the 2 in-plane directions only [29, 35], is used to compute the block elastic properties. Starting from a two-hole block configuration representing hollow concrete blocks, the block void ratio *e* is decreased by increasing the number of holes in two block directions 1 and 3, as defined in Fig. 5a. To resemble real vertically-perforated blocks, a ratio of two is kept between the hole number in the block directions 1 and 3, and the internal web thickness $t_{\rm int}$ is decreased (first row of blocks in Fig. 5a). In order to cover smaller void ratios, the number of holes is then kept constant while the internal web thickness is progressively increased (second row of blocks in Fig. 5a). The external shell is 15 mm thick for all blocks. Void ratios of less than 30%, which are clearly too low and therefore outside the practical range, are included only to visualise better the trends.

Expressions for the block elastic properties fitting the FEM analyses results are plotted in Fig. 5b, c:

17 $$\begin{aligned} \begin{aligned}&\frac{E_{{\rm B},1}}{E_{\rho }} = 1-e^{0.50} \quad \frac{E_{{\rm B},2}}{E_{\rho }} = 1-e \\&{\nu _{\rm {B,12}}} = \frac{E_{\rm {B},1}}{E_{\rm {B},2}}{\nu _{\rm {B,21}}} \quad \frac{\nu _{\rm {B,21}}}{\nu _{\rho }} = 1 \\&\frac{G_{\rm {B}}}{G_{\rho }} = 1-e^{0.56}, \end{aligned} \end{aligned}$$

where $E_{\rho }$, $G_{\rho }$ and $\nu _{\rho }$ are the properties of the isotropic material used for the blocks, such as concrete, clay, mud, stone, etc., and $G_{\rho }=E_{\rho }/(2(1+\nu _{\rho }))$. For a solid and therefore supposedly isotropic block, the block moduli equal the material moduli. For increasing void ratios, the block moduli reduce, with $E_{\rm B,2}$, the block modulus parallel to the holes, showing an expected linear decrease, and $E_{\rm B,1}$, the modulus perpendicular to the holes, decreasing more strongly in a non-linear fashion. Focusing on the ratios between the resulting block shear modulus $G_{\rm B}$, the Poisson’s ratios $\nu _{\rm B,12}$, $\nu _{\rm B,21}$ and the corresponding properties of the block material, $G_{\rho }$ and $\nu _{\rho }$ respectively, it can be noticed that, while $\nu _{\rm B,21}/\nu _{\rho }$ is seemingly not influenced by the block void ratio, $G_{\rm B}/G_{\rho }$ and $\nu _{\rm B,12}/\nu _{\rho }$ reduce non-linearly with *e*.

### Influence of vertically-perforated blocks on the masonry elastic properties

**Fig. 6:**
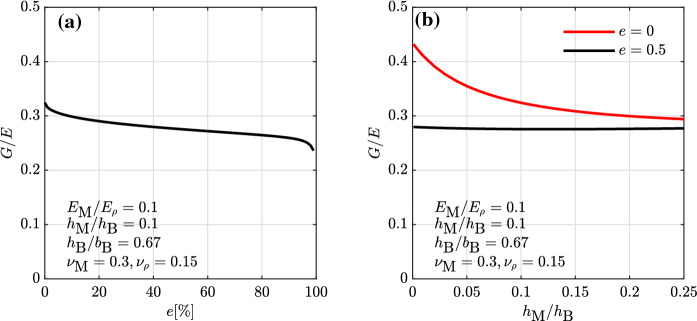
Influence of the block void ratio on the shear-to-elastic modulus ratio. Head and bed joints of equal thickness

Equation 17 are plugged into Eqs. 8 and 15 of the derived expressions for *E* and *G* in order to assess the influence of the block perforation on the masonry elastic properties. Figure 6a presents the development of the *G*/*E* ratio with increasing block void ratio *e*, illustrating that the ratio decreases with increasing block void ratio. For void ratios of, e.g. 25, 50 and 70% respectively, the *G*/*E* ratio decreases by about 10, 15 and 17% with respect to the value obtained for solid blocks. This holds for the set of parameter investigated in Fig. 6a. A further parametric study is included in Fig. 6b, where it appears that an increase in block void ratio leads to, first, a decrease in sensitivity of the *G*/*E* ratio to the joint-to-block height ratio and, second, a decrease of the *G*/*E* value which, for relatively thin joints, can be 2 times larger than the 15% shown in Fig. 6a. A decrease in sensitivity of the *G*/*E* ratio to the contrast, which is not shown in the figure, is also found for high void ratios.

### Influence of unfilled head joints on the masonry elastic properties

**Fig. 7:**
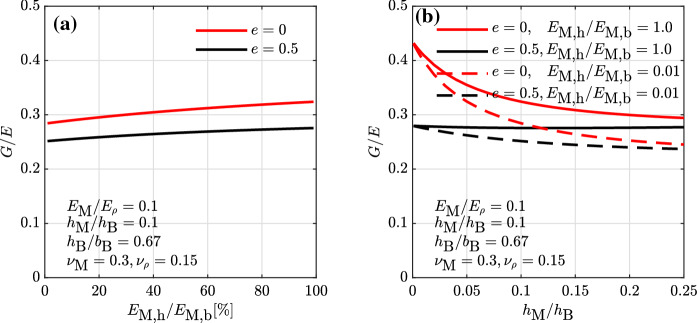
Influence of the head-to-bed joint elastic modulus ratio on the shear-to-elastic modulus ratio. Head and bed joints of equal Poisson’s ratio and equal thickness

The expressions for *E* and *G* derived in Sect. 4 have been obtained for head and bead joints sharing the same elastic properties (Eqs. 8, 15). The approach used for the derivation, however, allows for the attribution of different elastic moduli to each cell of the RVE. In this regard, assigning a different elastic modulus to the head joints, namely $E_{\rm M,h}$ to cells 2 and 5 of the RVE, and to the bed joints, namely $E_{\rm M,b}$ to cells 3, 4, 6, in Eqs. 2–5 enables the assessment of the influence of partly or fully unfilled head joints on the masonry elastic properties. The results of changing the ratio $E_{\rm M,h}/E_{\rm M,b}$ on the masonry *G*/*E* ratio are presented in Fig. 7 for solid blocks and blocks with a void ratio of 50%. For the investigated parameter set, the *G*/*E* ratio increases from unfilled to filled head joints, with about a 12% increase for solid blocks and a 10% increase for hollow blocks (Fig. 7a). Moreover, the decrease in *G*/*E* ratio with increasing joint-to-block height ratios already observed in Fig. 4 is more pronounced when the head joints are unfilled rather than when they are filled (Fig. 7b).

It is worth noticing that these results might, however, not show the full impact of unfilled head joints on the wall initial stiffness, as the used homogenization approach does not capture the influence of the relative rotations between the blocks. In a masonry wall with unfilled head joints loaded in shear and compression, the blocks undergo larger relative rotations leading to a further decrease in the wall stiffness. In order to seize this effect, a homogenization technique in the framework of a micropolar continuum theory could be used [22–24, 45–48]. The effect of the relative block rotations on the overall wall stiffness is found to become important when the size of the block is large compared to the size of the wall, i.e. for walls with effective lengths smaller than 7 to 5 times the block size [23, 24]. In most walls found in masonry buildings this effect is therefore supposed to be rather limited.

## Practical recommendations for the *G*/*E* ratio

The expressions for the masonry elastic properties obtained in this paper are used, first, to test the values for the *G*/*E* ratio proposed in the codes and the literature (Sect. 7.1), and, second, to propose new values of said ratio for different masonry typologies (Sect. 7.2).

### Comparison with codes and literature

As shown in Sects. 5 and 6, the shear-to-elastic modulus ratio *G*/*E* is influenced by the geometric and material parameters of the masonry wall and, for many configurations, this ratio differs from the value of 0.4 currently recommended by the codes [1–5].

The values for the *G*/*E* ratio vary quite significantly from a lower bound of about 0.15 to an upper bound of about 0.45. The upper bound value, along with the standardized value of 0.4, is reached only in the case of solid blocks and in the limit for increasingly thin mortar joints, that is for $e=0$ and $h_{\rm M}/h_{\rm B} \rightarrow 0$, which corresponds to the case of dry stacked masonry [27]. The range of 0.25–0.33 derived from the values for *E* and *G* proposed in the commentary [10] to the Italian technical standard NTC 08 [11] is more within the range of the obtained values, even though the standard does not capture the influence of parameters such as the block or joint Poisson’s ratio, the contrast, the block aspect ratio and the joint-to-block height ratio on the *G*/*E* ratio highlighted in the previous sections. The ratio of 0.13 calculated in Sect. 2 based on the proposal by Tomazevic [7] is close to the lower bound of the value of 0.15. However, as visible from Fig. 4, such a low value is only reached for very thick mortar joints, with $h_{\rm M}/h_{\rm B}> 0.25$, i.e. about five times thicker than the joints in the walls tested by Tomazevic [7]. As already indicated in Sect. 2, the source of such low experimental value for *G*/*E* can be related to the fact of correlating *G* to the wall stiffness measured at the onset of visible cracking, which may give a *G*/*E* that is approximately two times lower than the value that the expressions developed in this paper would predict for those tests.

**Fig. 8:**
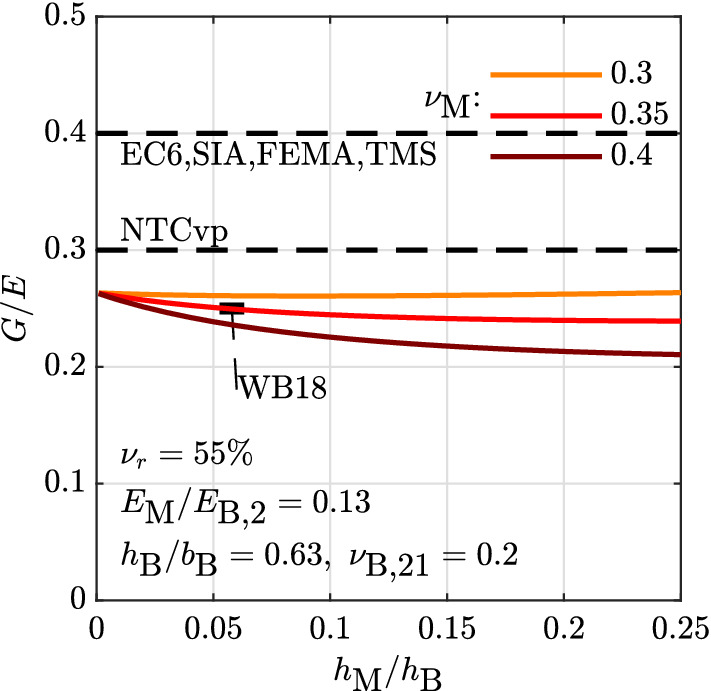
Comparison between *G*/*E* values predicted by the obtained expressions and code and literature provisions for hollow clay brick, cement-based mortar and filled head joint masonry walls tested by Petry and Beyer [13]: Part 1 of Eurocode 6 [1] (EC6), SIA 266 [4], FEMA 356 [2], TMS 402 [5], NZSEE [3], the value derived from the Italian technical standard NTC 08 [10, 11] for vertically-perforated blocks (NTCvp) and the value deduced by Wilding and Beyer [12] from the tests (WB18)

Figure 8 compares the *G*/*E* ratio derived from the obtained expressions with the *G*/*E* ratios obtained by other authors for shear-compression tests on modern hollow clay brick masonry walls [13]. For these tests, a ratio of $G/E=0.25$ was put forward [8, 12]. In those tests, the blocks dimensions were $h_{\rm B}=190$ mm, $b_{\rm B}=300$ mm [13], corresponding to $h_{\rm B}/b_{\rm B}=0.63$. Head and bed joints were of equal thickness, $b_{\rm M}=h_{\rm M}$, varying between 10 and 12 mm [13] which corresponds to $h_{\rm M}/h_{\rm B}=0.053-0.063$. $E_{\rm B,2}$, the brick elastic modulus perpendicular to the bed joints, is calculated as $E_{\rm B,2}=140 f_{\rm B,c}=4900$ MPa, with $f_{\rm B,c}=35$ MPa as the block compressive strength [13]. Assuming $E_{\rm B,2}$ and knowing that $E=3550$ MPa is the mean value obtained from simple compression tests made on masonry wallettes [13], $E_{\rm M}=616$ MPa is obtained when the mean of $h_{\rm M}$, 11 mm, is used. This corresponds to $E_{\rm M}/E_{\rm B,2}=0.13$. The void ratio of the vertically-perforated clay bricks is estimated at 55%, based on a photo of the brick [13], from which $E_\rho =10889$ MPa is obtained by Eq. 17. $\nu _{\rho }= \nu _{\rm B,21}$ is set equal to the masonry Poisson’s ratio of 0.2 [13], and, since $\nu _{\rm M}$ is not available [13], a range of values between 0.30 and 0.40 is tested. Plugging these values into the derived expression for *E* and *G*, *G*/*E* ratios between 0.24 and 0.26 are obtained (Fig. 8). This is in agreement with the average ratio of 0.25 deduced from the tests [8, 12]. The expressions predict $G/E=$0.25 for $\nu _{\rm M}=0.35$, which is a reasonable value for a mortar Poisson’s ratio.

### Recommendations for different masonry typologies

Values for *G*/*E* ratios are given for the following masonry typologies, listed from the most historical to the most recent one (Table 1): dry-stacked and mortared stone masonry made of dressed regular blocks; brick masonry made of clay and calcium silicate solid blocks and cement- and lime-based mortar; brick masonry made of hollow, or vertically-perforated, calcium silicate and clay blocks, the latter being with filled or unfilled head joints, and cement-based or thin layer mortar. The recommended values are derived by plugging into the analytical expressions for *E* and *G* typical values for the mortar-to-block elastic modulus ratio (contrast), the joint-to-block height ratio, the block aspect ratio and the block void ratio. These are all easily determinable parameters that allow for a better estimation of the *G*/*E* ratio in the practice. In order to account for uncertainties on the mortar Poisson’s ratio, which has a large impact on the *G*/*E* ratio and is not easily determinable from tests, the values for *G*/*E* provided in the table are rounded to 0.05.

As discussed in Sect. 7.1, the case of dry stacked masonry can be retrieved as the limit value for very thin joints and very stiff blocks [27], albeit without taking into account the complex contact phenomena at the block-to-block interface possibly influencing the elastic properties of this masonry typology as well. At this limit, according to the model, the *G*/*E* ratio depends on the block Poisson ratio only (Fig. 4). Taking $\nu _{\rm B}=0.15$ from uni-axial simple compression tests carried out on stone specimens and measured at the specimen mid-height [49], a ratio of 0.42 is obtained, which is finally rounded to 0.40.

A very low contrast, e.g. $E_{\rm M}/E_{\rm B} = 0.01$, is also representative of mortared stone masonry, composed of solid blocks whose stiffness is rather high compared to the lime-based mortar generally used in historical construction [49, 50]. For this typology, $\nu _{\rm B}=0.15$ and $\nu _{\rm M}=0.3$, as well as a block aspect ratio of 0.5 is assumed. The joint thickness typically varies between 0.01 and 0.1, for which the *G*/*E* ratio ranges between 0.32 and 0.26. A value of 0.30 is therefore recommended.

Solid brick masonry is characterized by a higher contrast than stone masonry. In the absence of indications on the $E_{\rm M}/E_{\rm B}$ ratio, the $f_{\rm c,M}/f_{\rm c,B}$ ratio is used to obtained typical values for this typology. For clay and calcium silicate bricks, $E_{\rm M}/E_{\rm B} = 0.1$ and 0.3 respectively [51]. Moreover, the joint thickness can be as high as 0.1 times the block thickness, while the block aspect ratio is usually around 0.25 and 0.3 respectively [51]. Using $\nu _{\rm B}=0.20$ and $\nu _{\rm M}=0.3$, $G/E=0.33$ and 0.37 are obtained, which are rounded to 0.35 and 0.40. Setting $E_{\rm M}/E_{\rm B}$ to 0.01 and keeping the other values unchanged, the case of solid clay bricks with lime-based mortar, which is also used in historical construction, is obtained, with $G/E=0.28$ rounded to 0.30.

Hollow bricks usually have a void ratio of about 50% and, when used with general purpose or lightweight mortar, a joint thickness of roughly 0.06 times the block height [7, 13]. The $E_{\rm M}/E_{\rm B}$ ratio is typically about 0.1 and the block aspect ratio is between 0.63 and 1 [7, 13]. For these values, and using $\nu _{\rm B}=0.20$ and $\nu _{\rm M}=0.3$, *G*/*E* ratios around 0.25 are obtained. For hollow bricks with unfilled head joints the ratio decreases to 0.20. The use of thin layer mortar bed joints for this typology makes the *G*/*E* ratio increase to 0.25. The same ratio is found for perforated calcium silicate bricks.

**Table 1:** Proposed values for the *G*/*E* ratio for different masonry typologies

Masonry typology	Void ratio *e* (%)	Contrast $E_{\rm M}/E_{\rm B}$	Joint thickness $h_{\rm M}/h_{\rm B}$	Block aspect $h_{\rm B}/b_{\rm B}$	*G*/*E*
Dressed regular dry stacked stone masonry	0	< 0.01	$\rightarrow$ 0	0.25–1	0.40
Dressed regular mortared stone masonry	0	0.01	0.01–0.1	0.5	0.30
Solid clay brick masonry, lime mortar	0	0.01	0.1	0.25	0.30
Solid clay brick masonry, cement mortar	0	0.1	0.1	0.25	0.35
Solid calcium silicate brick masonry	0	0.3	0.1	0.3	0.40
Perforated calcium silicate brick masonry	50	0.3	0.1	0.63–1	0.25
Hollow clay brick masonry	50	0.1	0.06	0.63–1	0.25
Hollow clay brick masonry, unfilled head joints	50	0.1	0.06	0.63–1	0.20
Hollow clay brick masonry, unfilled head joints, thin mortar bed joints	50	0.1	0.01	0.63–1	0.25

## Conclusions

This paper studies the ratio between the shear and elastic modulus *G*/*E* of in-plane loaded unreinforced masonry walls. Currently, design codes provide values based on historic convenience rather than scientific evidence. To improve the situation, analytical expressions for the elastic and shear moduli *E* and *G* of running-bond masonry walls were derived and validated against 3D FEM analyses.

Parametric studies using these expressions and assuming solid isotropic blocks show that the *G*/*E* ratio strongly depends on the joint-to-block elastic modulus ratio, the joint-to-block height ratio, and the joint Poisson’s ratio, while only showing a limited dependency on the block aspect ratio and the block Poisson’s ratio. Furthermore, investigating the effect of orthotropic vertically-perforated blocks and unfilled head joints on the masonry elastic properties shows that an increasing block void ratio can decrease the *G*/*E* ratio up to 30%, and unfilled head joints can decrease the ratio by about 15%. The studies show that the *G*/*E* ratio of 0.4 currently included in most international codes is too high for most masonry typologies. As a result, the shear deformations of masonry elements tend to be underestimated. The expressions derived in this paper allow for the accurate estimation of the elastic parameters *E* and *G* of masonry as well as its *G*/*E* ratio for different masonry configurations. Practical values for the *G*/*E* ratio are provided by these expressions based on the masonry typology classically found in existing buildings. The resulting *G*/*E* ratios vary, depending on the masonry typology, between 0.20 and 0.40.

## Reproducibility of the article content

Figures 1, 4, 5b, c, 6, 7 and 8 as well as the values provided in Table 1 of this paper can be reproduced with the code provided at the following link: 10.5281/zenodo.2590596. The files include: the implementation of all the presented analytical expressions for *E* and *G*, the results and input files from the 2D and 3D FEM analyses, the analytical expressions of the ‘Pan3D’ model, and an additional numerical study of the influence of varying RVE thicknesses on the accuracy of the introduced model.
